# Efferent Vestibular Neurons Show Homogenous Discharge Output But Heterogeneous Synaptic Input Profile *In Vitro*

**DOI:** 10.1371/journal.pone.0139548

**Published:** 2015-09-30

**Authors:** Miranda A. Mathews, Andrew Murray, Rajiv Wijesinghe, Karen Cullen, Victoria W. K. Tung, Aaron J. Camp

**Affiliations:** 1 Discipline of Biomedical Science, Bosch Institute, The University of Sydney, Sydney, New South Wales, Australia; 2 Department of Biochemistry and Molecular Biophysics, Howard Hughes Medical Institute, Columbia University, New York, United States of America; 3 Discipline of Anatomy and Histology, Bosch Institute, The University of Sydney, Sydney, New South Wales, Australia; McGill University, CANADA

## Abstract

Despite the importance of our sense of balance we still know remarkably little about the central control of the peripheral balance system. While previous work has shown that activation of the efferent vestibular system results in modulation of afferent vestibular neuron discharge, the intrinsic and synaptic properties of efferent neurons themselves are largely unknown. Here we substantiate the location of the efferent vestibular nucleus (EVN) in the mouse, before characterizing the input and output properties of EVN neurons *in vitro*. We made transverse serial sections through the brainstem of 4-week-old mice, and performed immunohistochemistry for calcitonin gene-related peptide (CGRP) and choline acetyltransferase (ChAT), both expressed in the EVN of other species. We also injected fluorogold into the posterior canal and retrogradely labelled neurons in the EVN of *ChAT*:: *tdTomato* mice expressing tdTomato in all cholinergic neurons. As expected the EVN lies dorsolateral to the genu of the facial nerve (CNVII). We then made whole-cell current-, and voltage-clamp recordings from visually identified EVN neurons. In current-clamp, EVN neurons display a homogeneous discharge pattern. This is characterized by a high frequency burst of action potentials at the onset of a depolarizing stimulus and the offset of a hyperpolarizing stimulus that is mediated by T-type calcium channels. In voltage-clamp, EVN neurons receive either exclusively excitatory or inhibitory inputs, or a combination of both. Despite this heterogeneous mixture of inputs, we show that synaptic inputs onto EVN neurons are predominantly excitatory. Together these findings suggest that the inputs onto EVN neurons, and more specifically the origin of these inputs may underlie EVN neuron function.

## Introduction

Our sense of balance is fundamental to our ability to interact with our environment, yet central nervous control of peripheral vestibular activity remains poorly understood. The vestibular labyrinth receives dual innervation: the afferent component relays information regarding linear and rotational motion of the head from the periphery to the brainstem, and the efferent division which originates in the brainstem and terminates on vestibular hair cells and primary afferents. These efferent neurons have been shown to exert direct inhibitory modulatory control over type II hair cells [1–3], and direct excitatory control of afferent nerve fibres contacting both type I and type II hair cells [4–6].

Vestibular efferent neurons have been identified in all vertebrates studied and are typically located within proximity to the genu of the facial nerve, although subtle variations exist depending on the species [7–9]. Dendritic morphology also varies across species such that mammals typically exhibit a bilateral organization of efferent projections whereas other vertebrates display predominantly unilateral projections [5, 10–12]. In comparison to afferent fibres only a small number of efferent neurons innervate the vestibular periphery [13]. However efferent neurons bifurcate extensively, allowing for a single efferent fibre to innervate more than one vestibular end organ and thus exert influence over hair-cell/afferent signalling in multiple planes of head motion [10, 13–16].

Previous work has described the effects of vestibular efferent activation on afferent sensitivity. For example, work in chinchilla and macaque has shown that electrical stimulation of efferent fibres results in increased background discharge rate of afferent fibres, particularly the irregularly discharging fibres [17, 18]. Similarly, work in toadfish has shown both increased afferent discharge in response to efferent stimulation, and a simultaneous decrease in afferent gain (i.e. reduced afferent sensitivity) [1, 4, 19].

Despite the observed effects of vestibular efferent activation, the underlying context of this activation remains speculative. Most studies have focussed purely on the impact of efferent activation on afferent signalling with little attention afforded to the intrinsic and synaptic influences on the central vestibular efferent neurons themselves [18, 20]. As such, single-cell recordings from efferent vestibular neurons under normal physiological conditions remain scarce. Indeed, only one study has attempted to characterise the intrinsic physiological properties of efferent vestibular nucleus neurons. Using a transgenic mouse model Leijon and Magnusson (2014) showed that efferent vestibular neurons display a relatively hyperpolarised resting membrane potential, a characteristic action potential discharge pattern in response to depolarising current injection, relatively low discharge rates, and low gain. The authors suggest that the distinctive discharge pattern and low gain of these neurons facilitates the regulation of both the fast and slow components of afferent response, and supports a feedback mechanism to mediate gravity-induced vestibular activity, respectively [21].

Here we significantly extend on this work to a larger population of efferent vestibular nucleus (EVN) neurons. We confirm the location of EVN neurons in the mouse brainstem using choline acetyltransferase (ChAT) and calcitonin gene-related peptide (CGRP) immunohistochemistry—both known to be expressed in EVN neurons [21–25], as well as retrograde tracing from the vestibular periphery. We then go further and describe the density of these neurons within the nucleus. Next we examine the passive membrane properties and discharge profile of efferent vestibular neurons, extending on previous work by including analysis of a subthreshold afterdepolarization (ADP). As a further means of identifying this group of neurons *in vitro* we also provide a comparison with neighbouring medial vestibular nucleus (MVN) neurons. Finally, we describe for the first time the synaptic input profile of efferent vestibular nucleus neurons using pharmacological blockade of excitatory and inhibitory neurotransmission under whole-cell voltage-clamp conditions. This work provides fundamental insight into the underlying mechanisms of central control of peripheral vestibular function.

## Material and Methods

### Animals and ethics statement

All experiments were performed using both male and female (3–5 weeks) C57Bl/6 mice. This study was carried out in strict accordance with the recommendations of the Animal Care and Ethics Committee of the University of Sydney (approved protocol number: K22/5.13/3/5983), and the Institutional Animal Care and Use Committee of Columbia University (approved protocol number: AC-AAAG8461). All efforts were made to minimize animal suffering.

### Tissue preparation

Mice were deeply anesthetised with an intra-peritoneal injection of ketamine (100 mg/kg) (Parnell Living Science, Australia) and decapitated. For electrophysiological recordings, the brainstem was exposed as follows: an incision was made in the skull at lambda, before the paired parietal bones were reflected, and the occipital bone and inner ears were removed posteriorly. The brainstem was continuously bathed *in situ* using ice-cold sucrose-modified artificial cerebrospinal fluid (sACSF) containing (in mM): 236 sucrose, 26 NaHCO_3_, 11 glucose, 3 KCl, 1.25 NaH_2_PO_4_, 2 MgCl_2_, 2.5 CaCl_2_. The sACSF was gassed with carbogen (95% O_2_, 5% CO_2_) to achieve a final pH of 7.2–7.3, ensuring tissue viability during electrophysiological recordings.

For electrophysiology the brainstem was isolated from its encasing bone and forebrain, and then mounted rostral end down on a polystyrene (Styrofoam) block glued to the stage of a vibrating microtome (DSK Microslicer DTK-1000, Kyoto, Japan) using cyanoacrylate glue (Selleys, Padstow, Australia). The microtome stage was transferred to a cutting chamber filled with ice-cold sACSF continuously perfused with carbogen gas. Transverse slices were cut at 200 μm and those containing the genu of the facial nerve (typically 2–3 slices in total) were collected in sACSF before being transferred to an incubation chamber containing ACSF (120 mM NaCl substituted for sucrose) at room temperature (21–23°C), and allowed to equilibrate until recording (typically 30 mins).

For immunohistochemistry, mice were prepared as described above for electrophysiology. However, once the brainstem was exposed, the cranial vault was immersion-fixed in 4% paraformaldehyde in 0.1 M phosphate buffered saline (PBS, pH 7.4) at 4°C over four nights. The brainstem was then removed from the cranial vault and cryoprotected overnight at 4°C in 30% sucrose in 0.1M PBS (pH 7.4). A CO_2_ freezing microtome (Leica, Germany) was used to make consecutive 40 μm transverse sections of the brainstem from the apex of the spinal cord through to the caudal end of the cerebellum. Sections were collected and stored in tissue culture wells filled with 0.1 M PBS until use. For each sectioned brainstem, consecutive series (10 sections of 40 μm thickness) that included the facial nerve were processed for immunohistochemistry.

### Immunohistochemistry

Following permeabilization in alcohol and blocking in peroxidase blocker (10% methanol, 0.3% H_2_O_2_), free-floating sections were incubated in goat polyclonal antibodies to CGRP (1:500, Abcam ab36001) and ChAT (1:500, Abcam ab101755) for two hours at room temperature. Bound antibody was visualised using the avidin-biotin-peroxidase complex detection system (ABC, Vector Laboratories) with 3, 3”-diaminobenzidine (Sigma-Aldrich, St Louis MO) as the chromogen. After labelling, the sections were coated in warm alcoholic gelatine (40% ethanol, 0.5 g gelatine) mounted onto gelatine-coated slides, air-dried at room temperature overnight, dehydrated in ethanol, cleared in xylene and cover-slipped with DPX (Sigma-Aldrich, St Louis MO). Consecutive series of sections labelled using antibodies to CGRP and ChAT were counterstained with cresyl violet (CV) and neutral red respectively before coverslipping.

Tissue sections were examined using a Leica DMLB microscope fitted with a ProgRes C14 camera (Jentopix, Jena Germany). The cell bodies of CGRP and ChAT positive neurons were identified on the basis of location and CGRP/ChAT labelling [21, 26]. The total unbiased cell number in the vestibular efferent nucleus was determined as follows: all nucleoli in CGRP/CV positive neurons were counted by eye using a 40x objective, focussing through the 40 μm sections to ensure the identification of the nucleolus. Nucleoli were counted in each of the consecutive sections in which the EVN appeared (~2–3 sections in total). Neuron size and the volume of the EVN was determined using FIJI analysis software [27]. Using a 40x objective, the nucleolus was brought into focus and photographed. The cells were traced with the polygon tool and the major and minor diameters of neurons (20 cells per nucleus) were determined using the ‘measure’ plugin in FIJI. For the volume of the EVN, the outline of the nucleus was traced in images of each section and the area measured. The total volume of the nucleus was determined as area multiplied by section thickness (40 μm) and the cell density as the number of cells/volume.

### Retrograde labelling

To identify cholinergic neurons projecting to the vestibular periphery, mice expressing Cre under the control of the ChAT promoter (*ChAT*::*cre*; [28]) were crossed to *ROSA-loxP-STOP-loxP-tdTomato* (Ai14; [29]) mice to generate *ChAT*:: *tdTomato* animals expressing tdTomato in all cholinergic neurons. To selectively label vestibular efferent neurons, P12 *ChAT*:: *tdTomato* mice were anesthetised with 3% isoflurane in oxygen, the area behind the ear was shaved and cleaned with iodine and isopropanol, and an incision was made 1 mm behind the right pinna. The muscles were separated and the posterior semicircular canal was visualised through the skull. A scalpel was used to thin the bone of the semicircular canal until a small (approximately 100 μm) hole was made. A pulled glass capillary (1.5 mm outer diameter; 1.12 mm inner diameter; 8 μm bore width; World Precision Instruments) was used to introduce 100 nl of 2% fluorogold (Sigma-Aldrich) in saline into the posterior canal over a period of 2 minutes, and the capillary left in place for one minute after injection. The hole was filled with bone wax, the skin incision sutured, and the animals given analgesics. Animals were sacrificed three days after injection by transcardial perfusion with 4% paraformaldehyde in phosphate buffer. The brains were removed and cryoprotected in 30% sucrose solution overnight. 50 μm sections were cut on a cryostat, and the floating sections were immunostained with a rabbit anti-fluorogold primary antibody (EMD-Millipore; 1:3000) and a FITC-conjugated secondary antibody (1:100; Jackson Immunoresearch). Sections were mounted on glass slides, coverslipped with mowiol (Sigma-Aldrich) and imaged using a Zeiss 510 confocal microscope.

### Electrophysiology

The slice with the best visible genu of the seventh cranial nerve was transferred to a small glass-bottom recording chamber and held down by a weighted net made from nylon strings fixed to a horseshoe-shaped flattened platinum wire. The chamber was continuously perfused with oxygenated ACSF at room temperature (21–23°C). Slices were observed using a fixed-stage microscope (Olympus BX-51W1, Tokyo, Japan) at low power (10x) to localize the EVN. A high power water immersion lens (40x) was used to visually identify individual efferent vestibular neurons based on their anatomical location—dorsolateral to the genu of the facial nerve. Micropipettes were fashioned from thin-walled borosilicate glass tubing (1.5 mm OD, Warner Instruments, Hamden, Connecticut) using a two-stage protocol on a micropipette puller (PP-830, Narishige, Tokyo, Japan) to achieve a final resistance of 3–5 MΩ. In order to characterise action potential and discharge properties, micropipettes were filled with potassium-based internal electrode solution containing (in mM): 70 potassium gluconate, 70 KCl, 2 NaCl, 10 HEPES, 4 EGTA, 4 Mg_2_-ATP, 0.3 Na_3_-GTP; with a final pH of 7.3 (adjusted using KOH). For characterizing miniature inhibitory, and excitatory post synaptic currents (mIPSCs and EPSCs respectively), pipettes were filled with a caesium-based internal electrode solution containing (in mM): 130 CsCl, 10 HEPES, 10 EGTA, 1 MgCl_2_, 2 MG_2_ATP, and 0.3 Na_3_GTP; with a final pH of 7.3 (adjusted u CsOH). Recording pipettes also contained 0.5 mg/mL Lucifer yellow (to allow for post-recording mapping of recording sites).

A motorised micromanipulator (MP-225, Sutter Instrument, California, United States) was used to manoeuvre the pipette within the slice. Recordings were targeted to the region dorsal to the genu of the facial nerve. A >1 GΩ seal (infinite resistance) was established between the micropipette and cell wall before negative pressure was applied via a suction port on the micropipette holder to establish the whole-cell configuration. All whole-cell current-clamp and voltage-clamp recordings were captured (20 kHz) using a Multiclamp 700B amplifier (Molecular Devices, Sunnyvale, California, USA). Data was acquired using Axograph X version 1.3.5 acquisition software (Axograph Scientific, Sydney, Australia) on an Intel-based Apple Macintosh iMac computer.

### Action potential and discharge properties

Recordings were made from cells with a healthy resting membrane potential between -49.6 and -64.6 mV. All membrane voltages were corrected for a calculated liquid junction potential of -4.6 mV. Series and input resistance were calculated from the response to a 10 mV hyperpolarizing voltage step from a holding potential of –70 mV at the beginning and end of each recording. Data were rejected if the series resistance changed by >20% during the course of an experiment. Pre-prepared protocols (hyperpolarizing and depolarizing steps, 20 pA amplitude over 1 s) were injected via the recording pipette and the responses used to characterize action potential (AP) and discharge properties of EVN neurons. Cells were classified as spontaneously active if they fired five or more action potentials at rest over 3 seconds. Action potentials were overlaid at their onset for both spontaneous and non-spontaneous cells and averaged to determine AP properties. APs were averaged from the first trace only for spontaneous cells or from the entire protocol excluding the first 200 ms following a stimulus for non-spontaneous cells. Spike amplitude was defined as the maximum membrane voltage (Vm) from the baseline (2.5 ms before the AP peak) and spike rise-time was defined as the time from 10% to 90% spike amplitude. Spike width was defined as the width determined at 50% of peak amplitude [30]. Afterhyperpolarization (AHP) amplitude was defined as the difference between baseline and the minimum membrane voltage of the trace. The afterdepolarization (ADP) was characterised by pharmacological blocakade using tetrodotoxin (TTX; 1 μM) and 3,5-dichloro-N-[1-(2,2-dimethyl-tetrahydro-pyran-4-ylmethyl)-4-fluoro-piperidin-4-ylmethyl]-benzamide (TTA-P2; 1 μM).

### Synaptic input profile

Excitatory postsynaptic currents (EPSCs) and miniature inhibitory postsynaptic currents (mIPSCs) were isolated using pharmacological blockade in whole-cell voltage-clamp mode using a CsCl-based internal solution (holding potential -70 mV). Series and input resistance was calculated and monitored in the same way as for current clamp recordings of AP and discharge properties above. Selective pharmacological blockers were added successively to the bath to isolate individual excitatory and inhibitory currents. mIPSCs were isolated by the addition of 6-cyano-7-nitroquinoxaline-2-3-dione (CNQX; 10 μM) and TTX (1 μM) which block EPSCs and action potential driven events resulting from AMPA/kainate glutamate receptors (GluR) and voltage-gated sodium channels respectively. Excitatory synaptic transmission was isolated by the addition of GABA_A_ receptor (GABA_A_R) antagonist bicuculline (10 μM), and glycine receptor (GlyR) antagonist strychnine (1 μM). Putative unclamped AP type events that had greater than 500 pA amplitude were not included in the analysis of EPSCs. Whilst rare, these were easily identified from the captured and overlayed events collected for each cell. At least 3 minutes of data were acquired following 2 minutes of exposure to the above blockers. Only one cell was recorded per slice to avoid incomplete washout of pharmacological agents. TTX was obtained from Alomone Laboratories (Jerusalem, Israel). All other pharmacological agents were obtained from Sigma Chemicals (St. Louis, MO).

Both mIPSCs and EPSCs were analysed off-line using a semi-automated, sliding template protocol within the Axograph analysis package that detected events above a specified threshold level while compensating for changes in recording noise between traces. Amplitudes of at least three times the noise SD (3σ) were accepted. mIPSCs and EPSCs detected by the template were individually assessed and accepted for analysis based on two criteria: events did not overlap, and records displayed a stable baseline (2.5 ms) prior to the rising phase and after the decay phase. Accepted events were aligned at their onset and averaged. Peak amplitude, rise time (calculated over 10–90% of peak amplitude), and decay time constant (calculated over 20–80% of the decay phase) were calculated within the Axograph analysis software.

### Data analysis

Student’s unpaired *t*-tests and ANOVA were used for comparisons between variables used to assess action potential and discharge properties, and mIPSC and EPSC characteristics. All data are presented as means ± SD. Statistical significance was set at *p*<0.05.

## Results

### Morphological characteristics of the EVN

**Fig 1:**
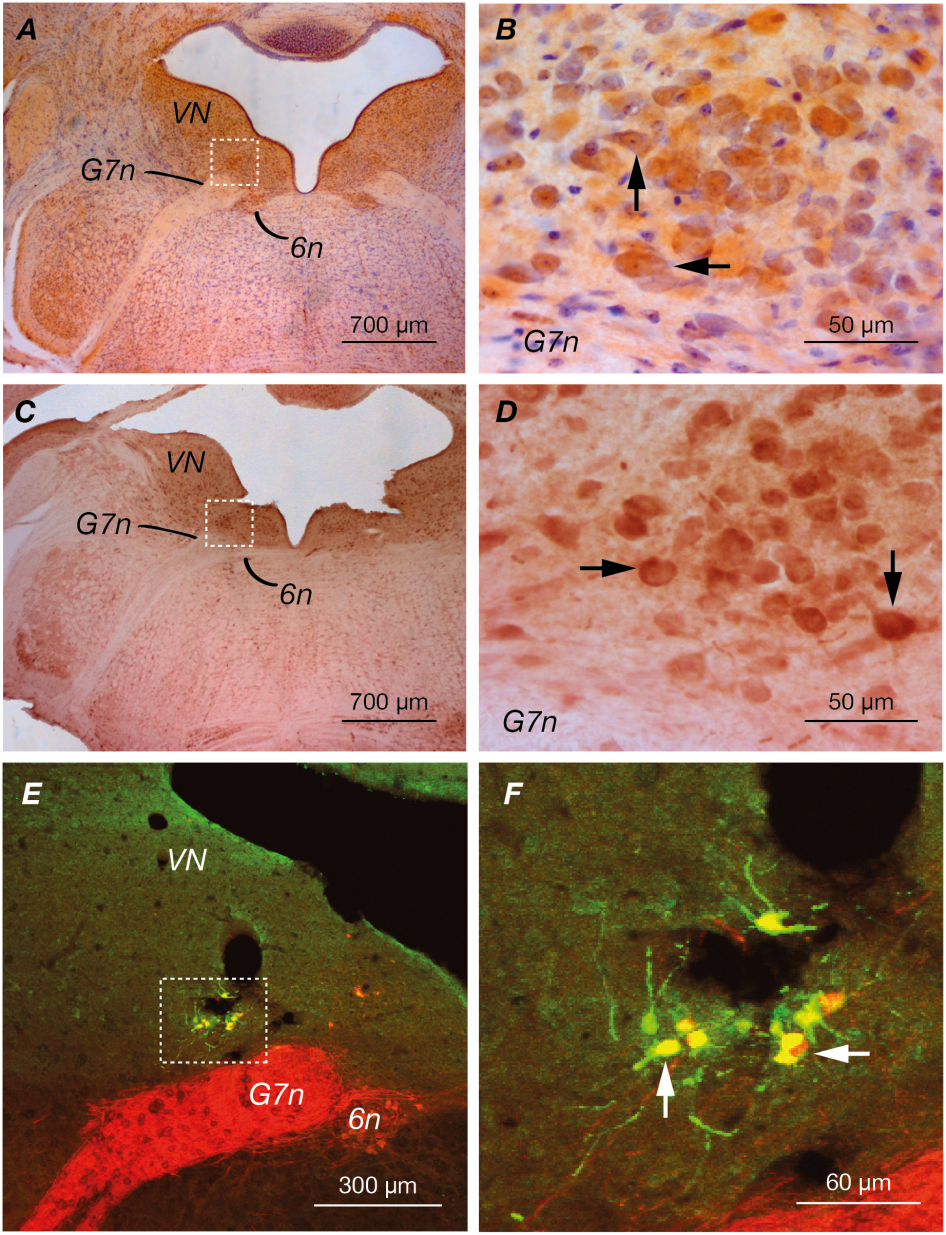
Immunohistochemical staining and retrograde tracing of the EVN. *VN*: vestibular nucleus; *G7n*: genu of seventh cranial nerve (facial nerve); *6n*: sixth cranial nerve nucleus (abducens nucleus). **(A&C)** Immunohistochemical staining for CGRP (*n* = 7) and ChAT (*n* = 4) respectively in transversely sectioned mouse brainstem. The EVN is located dorsolateral to genu of seventh nerve (box). Neurons in the abducens and vestibular nucleus were also labelled. **(B&D)** Higher power visualization of vestibular efferent nucleus. Arrowheads indicate CGRP and ChAT immuno-positive cells respectively. **(E&F)** Fluorogold injection into the posterior canal of *ChAT*:: *tdTomato* mouse strain under low and high power respectively (*n* = 4). Arrowheads indicate co-localization (yellow) of fluorogold (green) and tdTomato (red), confirming the location of vestibular efferent nucleus.

The location of the EVN was determined by immunohistochemical labelling using antibodies against CGRP and ChAT. Similar to previous studies in mice and rats [21, 25], small clusters of both CGRP (Fig 1A and 1B) (*n* = 7) and ChAT (Fig 1C and 1D) (*n* = 4) immuno-positive neurons were identified dorsolateral to the genu of the facial nerve (G7n). As expected ChAT and CGRP also labelled neurons in the abducens nucleus (6n), ventromedial to the genu of the facial nerve, as well as neurons in the vestibular nucleus (VN). The putative EVN location was confirmed by retrograde labelling from the peripheral vestibular apparatus. Fluorogold was injected into the posterior semicircular canal of *ChAT*:: *tdTomato* mice (*n* = 4). These mice express the red fluorescent protein tdTomato in cholinergic (ChAT positive) neurons (red). Fig 1E and 1F show that injected fluorogold (green) and ChAT positive tdTomato neurons are co-localised (yellow), confirming the location of the efferent nucleus. It is important to note that not all neurons in the posterior semicircular canal take up the flourogold, and as such only a subset of neurons in the EVN are labelled. No other fluorogold-expressing neurons were observed in the brainstem, confirming the specificity of the injections.

The total unbiased cell number in the EVN was determined by eye, focusing through 40 μm sections, counting nucleoli in consecutive sections in which the EVN appeared. The efferent nucleus is comprised of a small cluster of neurons (41.4 ± 5.2 neurons) per side (*n* = 5). Using FIJI analysis software, neuronal size and nucleus density was calculated. Neurons were circular in the centre of the nucleus and fusiform at the periphery (major axis 16.7 ± 3.3 μm; minor axis 9.4 ± 2.5 μm; circularity 0.7 ± 0.1). The volume of the nucleus was 1.61x10^-3^± 0.33x10^-3^mm^3^. Although the volume of the EVN varied between animals, right and left volumes were remarkably similar, with a difference of 0.1–1% between sides.

### Passive membrane and action potential properties

**Fig 2:**
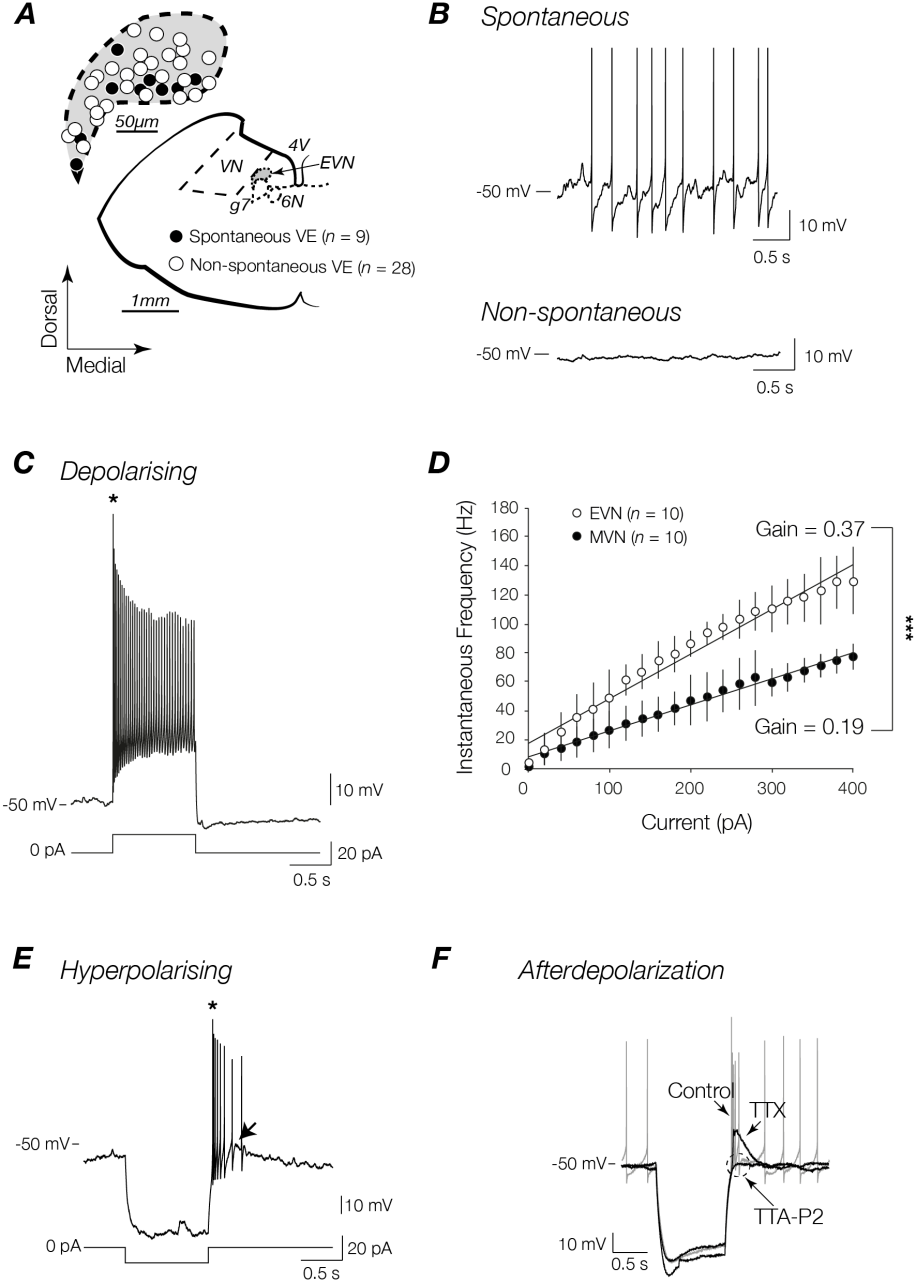
Discharge profiles of spontaneous and non-spontaneous EVN neurons. **(A)** Schematic view of transversely sectioned mouse brainstem. Inset shows map of recording sites from a subset of EVN neurons (37/54 recorded neurons). *VN*: vestibular nucleus; *G7n*: genu of seventh cranial nerve (facial nerve); *6n*: sixth cranial nerve nucleus (abducens nucleus); *4V*: fourth ventricle; *EVN*: efferent vestibular nucleus. **(B)** EVN neurons are either spontaneous firing (*n* = 16) (*top trace*) or non-spontaneously firing (*n* = 38) (*bottom* trace) at resting membrane potential and display homogenous discharge profiles in response to depolarizing **(C)** and hyperpolarizing **(E)** step currents. EVN neurons respond with a short burst (*) of high frequency action potentials (AP) at the onset of a depolarizing stimulus or the cessation of a hyperpolarizing stimulus. **(D)** Comparison of instantaneous frequencies as a function of injected depolarizing current from a subset of MVN and EVN neurons from which the slope of linear fit was used to calculate the gain of each neuron. *** *p*<0.001. **(F)** EVN neurons displayed an afterdepolarization (ADP) following release from inhibition (arrow in **(E)**). The ADP was mediated by T-type calcium channels—TTX (1 μM) abolished all APs, and TTA-P2 (1 μM) abolished the remaining response.

Fifty-four EVN neurons and twenty-two MVN neurons were recorded from mouse brainstem slices using the whole-cell current-clamp configuration. To confirm their location within the EVN individual EVN neurons dialysed with Lucifer yellow (Sigma Chemicals, St. Louis, MO) were imaged under fluorescent light. Of the fifty-four EVN neurons recorded, the locations of thirty-seven EVN were mapped onto a schema from the Paxinos and Franklin mouse brain atlas (plate 79 [31]) (Fig 2A). While post-recording site mapping was unavailable for the remaining EVN neurons, all displayed stereotypical passive membrane and action potential properties, as well as discharge profiles, and were thus included for further analysis. Since the EVN is a small nucleus no attempt was made to investigate the properties of neurons in the rostrocaudal extent of the EVN. In our sample of EVN neurons, 30% (16/54) were identified showed irregular spontaneous discharge (3.8 spikes/s), while the remaining 70% (38/54) were not spontaneously active (Fig 2B). Importantly, no differences were observed in the resting membrane potential of spontaneous and non-spontaneous subsets of EVN neurons (-58.2 ± 6.2 vs. -55.8 ± 7.1 mV respectively). Passive membrane and action potential properties of spontaneous and non-spontaneous EVN neurons, as well as neighbouring MVN neurons are compared in Table 1. When passive membrane properties of spontaneous and non-spontaneous EVN neurons were compared no significant differences were observed (Table 1). When compared with MVN neurons only one significant difference was observed. All EVN neurons showed significantly lower capacitance than neighbouring MVN neurons (*p*<0.01 and *p*<0.001 respectively; Table 1), a feature that suggests different neuronal morphologies that could help delineate the two vestibular nuclei.

**Table 1:** Summary of passive membrane and action potential properties of spontaneous EVN, non-spontaneous EVN, and MVN neurons.

Properties	Spontaneous EVN neurons (*n* = 16)	Non-spontaneous EVN neurons (*n* = 38)	MVN neurons (*n* = 22)
*RMP (mV)*	-58.2 ± 6.2	-55.8 ± 7.1	-56.6 ± 4.1
*Rs (MΩ)*	9.0 ± 2.9	11.5 ± 5.4	10.1 ± 5.6
*Ri (MΩ)*	442.7 ± 194.6	481.1 ± 217.7	393.7 ± 270.7
*Cm (pF)*	14.0 ± 3.2 **	12.1 ± 3.3 ^†††^	18.2 ± 5.8 ** ^†††^
*AP peak (mV)*	51.3 ± 10.8 **	52.0 ± 14.0 ^†††^	63.2 ± 9.9 ** ^†††^
*AP rise time (ms)*	0.6 ± 0.4	0.8 ± 0.3 ^†††^	0.4 ± 0.2 ^†††^
*AP half-width (ms)*	1.3 ± 0.5	1.5 ± 0.4 ^†††^	1.1 ± 0.4 ^†††^
*AHP (mV)*	-23.1 ± 3.5 ^‡‡‡^	-15.9 ± 7.7 ^‡‡‡^ ^†††^	-23.3 ± 5.8 ^†††^

All data are presented as means ± SD.

** *p*<0.01 between spontaneous EVN neurons and MVN neurons

^†††^ *p*<0.001 between non-spontaneous EVN neurons and MVN neurons

^‡‡‡^ *p*<0.001 between spontaneous and non-spontaneous EVN neurons

Comparisons made using student’s unpaired *t*-test.

When the action potential properties of spontaneous and non-spontaneous neurons were compared only one significant difference was observed. Interestingly spontaneous EVN neurons displayed significantly deeper afterhyperpolarization (AHP) when compared with non-spontaneous neurons (-23.1 ± 3.5 vs. -15.9 ± 7.7 mV, *p*<0.001), although this could be due to altered channel kinetics during the current injection required to generate action potentials in non-spontaneous neurons. Importantly, both spontaneous and non-spontaneous EVN neurons showed slower action potential kinetics than MVN neurons. The difference was most pronounced in the non-spontaneous EVN subset, which showed slower rise times (0.8 ± 0.3 vs. 0.4 ± 0.2 ms, *p*<0.001) and half-widths (1.5 ± 0.4 vs. 1.1 ± 0.4 ms, *p*<0.001) when compared with neighbouring MVN neurons. Non-spontaneous EVN neurons also showed smaller AHP amplitudes (-15.9 ± 7.7 vs. -23.3 ± 35.8 mV, *p*<0.001), although as mentioned above, this is potentially due to the current pulses used to generate action potentials in these neurons. In addition, all EVN neurons showed significantly smaller peak spike amplitudes than MVN neurons (51.3 ± 10.8 and 52.0 ± 14.0 vs. 63.2±9.9 mV, *p*<0.001).

### Discharge properties

EVN neurons responded to depolarizing current steps in a stereotypical way. All EVN neurons fired a short, high frequency burst of action potentials (asterisk in Fig 2C) that adapted rapidly over time at the onset of the depolarizing stimulus. Fig 2D shows the averaged instantaneous frequency of a sample of EVN (*n* = 10) and MVN (*n* = 10) neurons plotted as a function of injected depolarizing current amplitude. The slope of a linear fit was used to calculate the average gain across the subset of neurons. EVN neurons displayed a significantly higher gain, i.e. they are more sensitive to injected current [32] when compared with neighbouring MVN neurons (0.37 vs. 0.19 Hz/pA, *p*<0.001). Since no differences were observed in passive membrane properties of EVN neurons and MVN neurons including input and series impedance, the differences in gain are independent of intrinsic membrane properties and/or recording conditions [33].

In a similar way to that described for depolarizing current injection, EVN neurons responded to release from hyperpolarizing current steps with a characteristic high frequency burst of action potentials superimposed on a 6.7±7.8 mV (range 1.4 to 20.2 mV) afterdepolarization (ADP) (arrow in Fig 2E). This ADP increased with successively higher current amplitudes and then plateaued (not shown). Fig 2F shows the pharmacological dissection of the ADP. The addition of voltage-dependent sodium channel blocker TTX (1 μM) completely abolished EVN neuron discharge unmasking the underlying ADP. The addition of the selective T-type calcium channel blocker TTA-P2 (1 μM) abolished the ADP, confirming T-type calcium channels as the underlying mechanism for this discharge property. Interestingly, this calcium dependent feature is similar to that observed in other neurons implicated in gain modulation functions including thalamocortical relay cells in the lateral geniculate nucleus [34].

### Synaptic input profile

**Fig 3:**
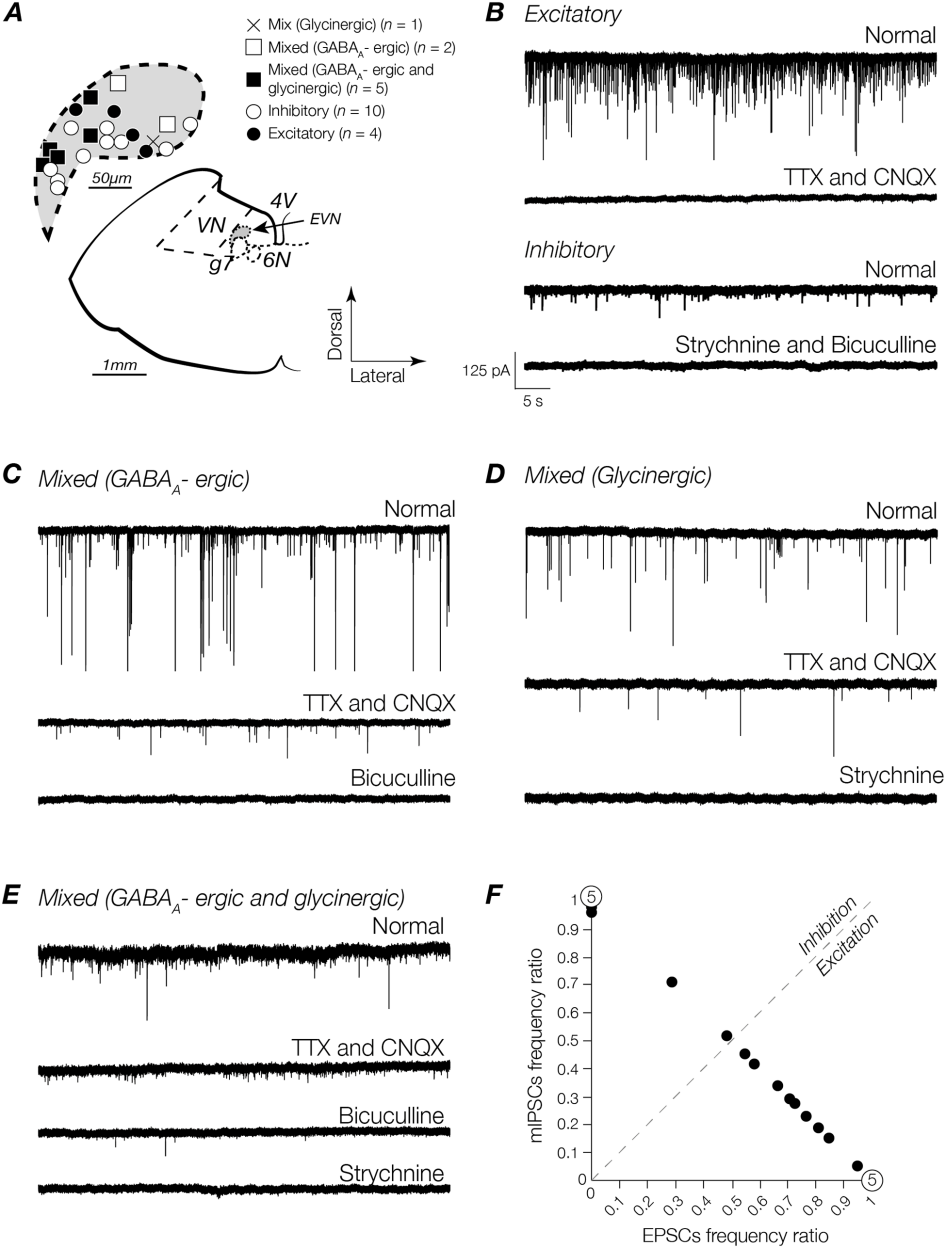
Identification and classification of excitatory and inhibitory profiles in EVN neurons. **(A)** Schematic view of transversely sectioned mouse brainstem. Inset shows map of recording sites (22/23 recorded neurons). *VN*: vestibular nucleus; *G7n*: genu of seventh cranial nerve (facial nerve); *6n*: sixth cranial nerve nucleus (abducens nucleus); *4V*: fourth ventricle; *EVN*: efferent vestibular nucleus. **(B)** *Top trace*: EPSCs recorded under normal conditions before the addition of drugs. *Second trace*: addition of CNQX (10 μM) and TTX (1 μM). *Third trace*: mIPSCs recorded under normal conditions before the addition of drugs. *Bottom trace*: addition of strychnine (1 μM) and bicuculline (10 μM) abolished all synaptic activity. Some neurons received excitatory inputs in conjunction with: GABA_A_R-mediated events **(C)** *Bottom trace*: addition of bicuculline to the bath abolished activity remaining after the addition of TTX and CNQX (*second trace*); GlyR-mediated events **(D)** *Bottom trace*: addition of strychnine abolished remaining activity following the addition of TTX and CNQX (*second trace*). **(E)** Other neurons received a combination of mIPSCs in addition to EPSCs. In these neurons, the addition of bicuculline reduced the frequency of synaptic activity (*third trace*) that was abolished by addition of strychnine (*bottom trace*). Scale bar in **(B)** is the same for all traces. **(F)** Frequencies of EPSCs and mIPSCs per cell calculated over a period of 30 seconds under the influence of excitatory and inhibitory synaptic activity blockers.

To investigate the contribution of glutamate-, GABA_A_-, and glycine-receptor mediated EPSCs and mIPSCs to the overall synaptic input profile of EVN neurons, whole-cell patch-clamp recordings were made in voltage-clamp configuration. As described above for action potential and discharge properties, each neuron (except one where mapping was unavailable), was mapped onto a standard schematic from the mouse brain atlas (Fig 3A). Recordings were made from 23 EVN neurons across the dorsoventral extent of the EVN in the presence of TTX (1 μM) and CNQX (10 μM) or bicuculline (10 μM) and strychnine (1 μM), to isolate inhibitory and excitatory synaptic activity respectively. Fig 3B shows that in a subset of neurons, addition of TTX and CNQX abolished all activity (top two traces). Conversely, in some neurons addition of bicuculline and strychnine abolished all activity (bottom two traces). These subsets were classified as exclusively excitatory or exclusively inhibitory expressing neurons respectively (Fig 3B).

For the remaining EVN neurons, the initial pharmacological blockade with TTX (1 μM) and CNQX (10 μM) reduced the frequency of synaptic events without abolishing activity. To determine which receptors the remaining synaptic events were mediated by, bicuculline (10 μM) and strychnine (1 μM) were introduced to the bath sequentially. In some neurons, the addition of bicuculline abolished all remaining synaptic activity (Fig 3C). In other neurons, the addition of strychnine (1 μM) instead abolished all remaining synaptic activity (Fig 3D). These neurons expressed exclusively GABA_A_R or GlyR mIPSCs respectively. In a subset of neurons, addition of bicuculline (10 μM) decreased the frequency of inhibitory synaptic input with the remaining activity abolished with the subsequent introduction of strychnine (1 μM), indicating a combination of GABA_A_R- and GlyR-mediated mIPSCs (Fig 3E). These neurons were classified as receiving a mixture of excitatory, and exclusively GABA_A_ergic or glycinergic inputs, or a combination of GABA_A_ergic and glycinergic mIPSCs; hereafter termed mixed (GABA_A_ergic), mixed (glycinergic), and mixed (GABA_A_ergic and glycinergic) respectively.

From the sample of 23 EVN neurons, 22% (5/23) received exclusively excitatory inputs while 30% (7/23) received exclusively inhibitory inputs. 13% (3/23) received excitatory input plus a mixture of GABA_A_ergic and glycinergic input, 30% (7/23) received excitatory input plus GABA_A_ergic input, and 4% (1/23) received excitatory input plus glycinergic input. Despite a heterogeneous synaptic input profile, Fig 3F shows that excitatory synaptic inputs predominate. In the subset of EVN neurons that receive a mixture of both excitatory and inhibitory inputs, the ratio of excitation to inhibition expressed as the function of excitation/total to inhibition/total is pushed heavily towards excitation (right of the line of unity). Of the mixed neurons encountered 69% (9/13) were skewed in this way (Fig 3F).

**Fig 4:**
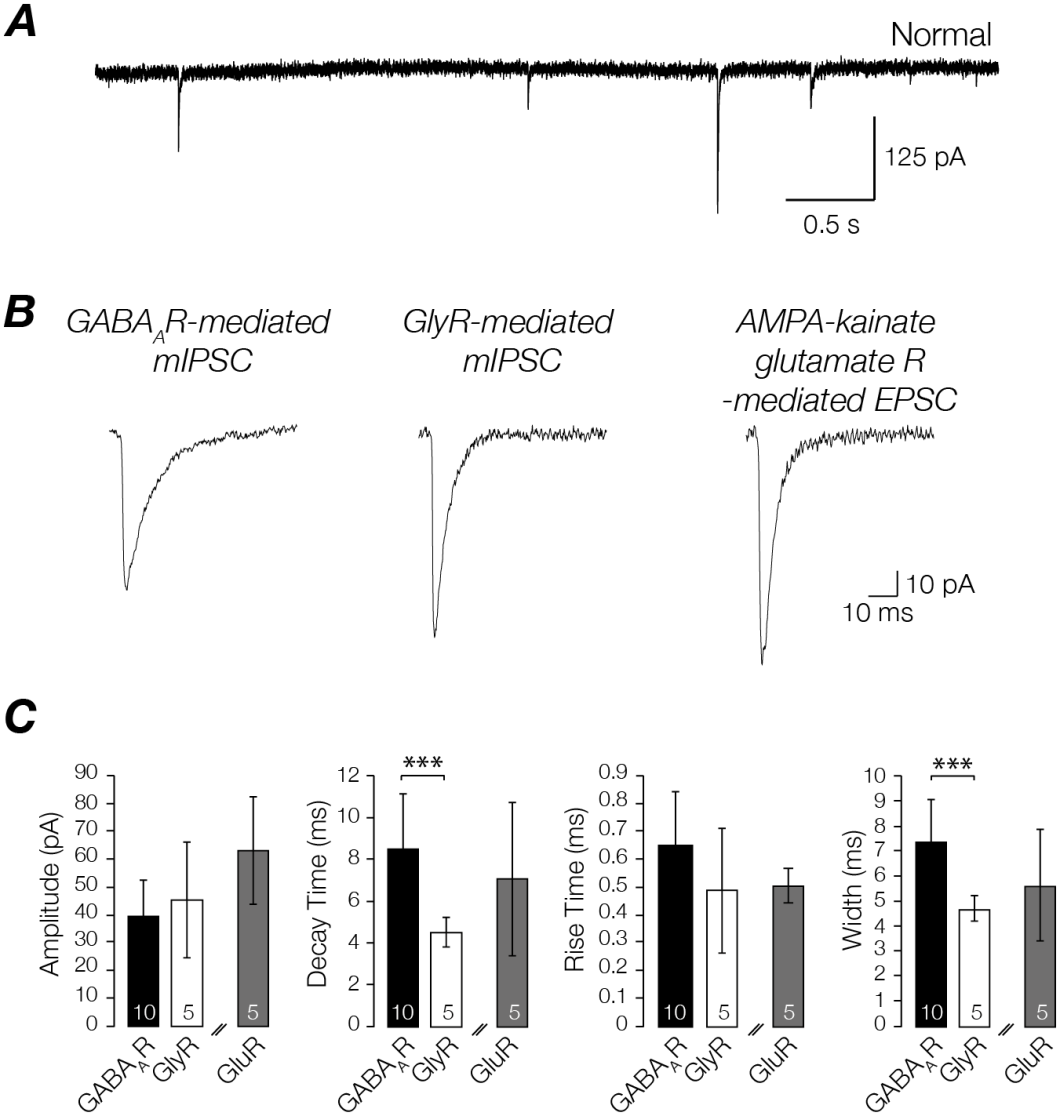
EPSC and mIPSC properties. **(A)** AMPA/kainate type glutamate receptor, GABA_A_R and GlyR-mediated EPSCs and mIPSCs. **(B)** Averaged GABA_A_R- and GlyR- mediated mIPSCs, and AMPA/kainate glutamate receptor mediated EPSC, isolated from the recordings shown above. **(C)** Bar graphs showing GABA_A_R-, GlyR-mediated mIPSCs and AMPA/kainate glutamate receptor mediated EPSC amplitude, decay time, rise time, and width. * *p*<0.05, ** *p*<0.01. Values within bars indicate the number of cells sampled. Double diagonal lines indicate that EPSC and mIPSCs values are not compared, but are presented on same bar graph for ease of demonstration.

The properties of GABA_A_R- and GlyR-mediated mIPSCs recorded from EVN neurons are compared in Fig 4. GABA_A_R- and GlyR-mediated mIPSCs were isolated via pharmacological blockade for analysis. The kinetics between inhibitory receptors differed significantly. GABA_A_R-mediated mIPSCs had slower decay times (8.6 ± 2.6 vs. 4.5 ± 0.7 pA, *p*<0.001) than GlyR-mediated mIPSCs. These kinetics are consistent with those reported for other neurons in the vestibular nuclei [33]. The kinetics of GluR-mediated EPSCs (both spontaneous and action potential driven) are shown alongside those of GABA_A_R- and GlyR-mediated mIPSCs in Fig 4.

## Discussion

### Morphological characteristics of the EVN

Vestibular efferent neurons have previously been identified dorsolateral to the genu of the facial nerve in mammals [35, 36]. Prior to this report, only one study has identified the location of these neurons in the mouse [21], although in this work no direct connectivity between the vestibular periphery in the labelled EVN was shown. In order to investigate the intrinsic and synaptic electrophysiological properties of efferent vestibular nucleus neurons we sought to confirm the location of the EVN using CGRP and ChAT immunohistochemistry as well as direct retrograde tracing from the vestibular periphery. Similar to Leijon and Magnusson (2014), we identified the EVN as a small region dorsolateral to the genu of facial nerve, and the number of neurons forming the nucleus was small, particularly in comparison to neighbouring vestibular nuclei where the number is ten-fold higher [37]. The small number of neurons is not surprising since previous work has shown that while efferent vestibular neurons make contact with both major vestibular afferents and type II hair cells directly, these connections are extremely divergent, i.e. a single efferent fibre makes contact with many afferents and hair cells in the vestibular periphery [10, 14, 38].

### EVN neuron passive membrane and action potential properties

Only one other study has measured the intrinsic properties of EVN neurons, and even so the responses of only nine neurons were reported. Using a transgenic mouse model, Leijon and Magnusson (2014) demonstrated that efferent vestibular neurons display a resting membrane potential of -76.49 ± 2.68 mV, pronounced AHPs, relatively low discharge rate, and low gain. In our larger sample of EVN neurons (*n* = 54) we found similar intrinsic electrophysiological properties to those reported by Leijon and Magnussen (2014) with a few exceptions. While we found no difference in input impedance, we did find a much lower capacitance. In addition, efferent vestibular neurons in our report showed faster action potential kinetics and more depolarized resting membrane potentials. Moreover, in our study we observed EVN neurons with two discharge profiles at rest—spontaneously active, and non-spontaneously active neurons (70% of the recorded population). While Leijon and Magnussen (2014) did not report distinct discharge profiles at rest, this is presumably a function of the very hyperpolarised resting membrane potential reported in their study. In addition, it is possible that these differences in discharge profiles are unmasked within our larger sample. Indeed, slow, irregular spontaneous activity of 4–5 spikes/s was previously reported in EVN neurons in the toadfish [39]. Despite this distinction in our report all EVN neurons had low discharge frequency at rest, consistent with the observations reported by Leijon and Magnusson (2014). For the spontaneously active EVN neurons in our study, this property was regulated by a relatively pronounced AHP (23.1 ± 3.5 mV), similar in amplitude to that reported by Leijon and Magnusson (2014). Since both spontaneous and non-spontaneous EVN neurons displayed similar passive membrane properties (including input impedance, and capacitance) the differences in AHP amplitude and discharge profile are presumably independent of recording conditions [33].

### EVN neuron discharge properties

All EVN neurons showed the same discharge profile—a short burst of high frequency APs at the onset of a depolarizing stimulus and cessation of a hyperpolarizing current (asterisk in Fig 2C) usually followed by sparse firing. This profile, characterized by an initial short latency discharge, was a feature also reported by Leijon and Magnusson (2014) who reported that upon depolarization, EVN neurons fired a single onset spike followed by sparse firing. This is an important point to note given that the location of the EVN lies in close proximity to the MVN, and that neighbouring MVN neurons are characterized by tonic discharge [30, 33, 40] at rates closer to 10–20 spikes/s [30, 41], suggesting that this may be a useful distinguishing feature for other studies targeting this nucleus. While tonic firing codes for the intensity of inputs, burst firing has been suggested to be advantageous in improving stimulus detectability and enhancing cortical activation, serving as a “wake-up call” during quiescent periods and in response to novel stimuli [42, 43]. For example, Weyand et al. showed that in the cat LGN during wakefulness (when tonic firing is the dominant discharge pattern) burst firing can be attributed to novel visual stimulation [44]. It is plausible for EVN neurons to aid in quick vestibular accommodation by generating transient bursts of APs in response to varying synaptic inputs that serve to modulate peripheral targets and adjust subsequent behaviour. One suggestion for the mechanism underlying a burst discharge profile is that the initial high frequency transient APs are related to the slow AHP (sAHP) phenomenon produced by voltage-dependent Ca^2+^-activated potassium currents that ultimately shape firing patterns (for a review see [45]). Slow afterhyperpolarizations have been observed throughout the CNS, occurring only after burst firing of APs [46–48], and as such may underlie this feature of EVN neuron discharge. Another possibility is that burst firing is generated following sufficient hyperpolarization to trigger a low threshold all-or-none Ca^2+^ spike [49] mediated by T-type Ca^2+^ channels. Cations moving through these channels have also been implicated in modelling firing patterns [50]. Similar to thalamocortical relay cells described in the mouse LGN [34], EVN neurons also show calcium-dependent ADP that contributes to burst firing (see Fig 2F). Although Leijon and Magnusson (2014) reported a subtle shoulder at the onset of depolarization similar to our observations, they did not show a measurable ADP following release from hyperpolarization. The reason for this absence is not clear, although as described above it is possible that this feature was unmasked in our larger sample of EVN neurons, or that the relative hyperpolarized membrane potential reported by Leijon and Magnusson (2014) obscured this feature. As described in our results it is interesting to note that in our sample, the amplitude of the ADP was variable, possibly contributing to the difference between the two studies.

We also compared EVN neuron and MVN neuron sensitivity to changes in the strength of their inputs (i.e. gain) and showed that EVN neurons are significantly more sensitive to input than MVN neurons. It is important to note though that for a small subset of non-spontaneously active EVN neurons, the gain is measured only from a small number of action potentials at the start of a stimulus due to the paucity of tonic firing in these neurons. Despite this, we feel that using the responses measured over a smaller amount of time is still a useful approximation, as there is little to no spike firing adaptation over the course of more prolonged stimuli such as those used to calculate gain in MVN neurons. Thus EVN neurons appear to be more suited to responding rapidly to alterations in synaptic strength without the requirement to code for activity in the temporal domain, i.e. how long the synaptic barrage continues [51].

### Synaptic profile of EVN neurons

The present study is the first electrophysiological characterization of the synaptic input profile of EVN neurons. The data from mouse brain stem slices showed that 52% of recorded EVN neurons received either exclusively excitatory (mediated by AMPA/kainate glutamate receptors) or inhibitory (mediated by GABA_A_ and glycine receptors) synaptic inputs. The remaining neurons displayed a mixed input profile receiving excitatory with inhibitory inputs in combinations of either: GABA_A_ergic and glycinergic together (13%), solely GABA_A_ergic (30%), or solely glycinergic (4%). In light of the observation that some EVN neurons fire spontaneously while others do not, it is tempting to question whether these discharge profiles relate to the excitatory and inhibitory input profiles observed here. While this idea remains a possibility, we suggest that it is unlikely given that the rate of spontaneous discharge observed here is very low, and could not in most cases be considered tonic—unlike MVN neurons, [30, 33, 40]. Though it is no surprise that GABA_A_-mediated synaptic inhibition dominates in the mouse EVN, as it does in neighbouring mouse MVN neurons [33], it is possible, based on the observed discharge profile of EVN neurons and work in vestibular afferents in the periphery [52], that GABA-ergic activity primarily acts to modulate fast glutamatergic excitation at the central EVN synapse. Indeed, it remains important to note that within mixed neurons, the frequency of excitatory activity mediated by AMPA/kainate glutamate receptors substantially overshadows the frequency of inhibition (64% vs. 9% respectively). Together these data suggest that the output of EVN neurons is predominately governed by combined excitatory drive from other parts of the CNS.

### Receptor subunit composition

When comparing inhibitory receptors, GABA_A_R-mediated mIPSCs exhibited slower kinetics (including rise and decay times) than GlyR-mediated mIPSCs. These results are similar to those reported for other rodent CNS regions where GABA_A_R- and GlyR-mediated mIPSCs have been compared [53–57], as well as other vestibular nuclei recorded under similar conditions [33, 58–60]. When combined with recent in situ hybridization and protein expression studies, some insight into the subunit composition of relevant receptors in mouse EVN can be gained. The available data in mice suggests that GABA_A_Rs are composed of α1, α2, α3, α4, γ2 δ subunits [61, 62], whereas GlyRs are composed of α1 and β subunits [63, 64]. Although studies in rat vestibular nuclei show that GluRs are composed of R1, R2/3 and R4 subunits [65, 66], studies that isolated GluR EPSC activity were performed in chicks [67, 68]. To our knowledge, there are no studies in AMPA/kainate glutamate receptor subunit composition in mice.

### Origin of synaptic inputs

Our results demonstrate that AMPA/kainate GluRs contribute to fast excitatory synaptic transmission, and GABA_A_Rs and GlyRs contribute to fast inhibitory synaptic transmission in mouse EVN neurons. The complete map of these inputs however, is unclear. EVN neurons have been shown to receive inputs as part of a feedback pathway—efferent endings terminate on afferent fibres as well as directly on type II hair cells and receive reciprocal innervation from these afferent fibres [69]. More distal sources presumably also contribute to the synaptic profile of EVN neurons. For example, viral transneuronal tracing revealed inputs from the hypothalamus, amygdala, and motor cortices amongst other areas [70]. Finally, local circuitries including contralateral projections from the opposite EVN, and other vestibular nuclei are also presumably a source of synaptic inputs onto EVN neurons. Indeed retrograde tracing in rats using microspheres identified neurons in the ipsilateral medial, lateral and superior vestibular nuclei projecting into the EVN [71]. Despite this information, it remains to be seen where the synaptic inputs measured here originate, and whether these inputs share defined electrophysiological properties.

### Functional implications

The data presented here show that mouse EVN neurons display diverse excitatory and inhibitory input profiles; yet use this information to produce a characteristic, homogeneous discharge output including a high frequency burst of APs at the cessation of a hyperpolarizing stimulus or the onset of a depolarizing stimulus. As such it is tempting to suggest that the drivers of the EVN are potentially more important than the final output i.e. the control of when the EVN is activated, or under what specific set of conditions the EVN is stimulated, is the defining feature of central control of peripheral vestibular sensitivity.

It has been shown that efferent vestibular fibres respond to stimulation of vestibular end organs including the canals [72–74] and otolith organs [75], and to non-vestibular stimuli such as epidermal pressure and limb movement [72, 76]. While the mechanisms are not well established, common to most discussions of vestibular efferent function is the suggestion that efferent neurons modulate peripheral vestibular activity by way of efferent-mediated excitation of irregular afferent discharge [3–6, 77, 78] (but see [79]). For example, work in chinchilla demonstrated efferent mediated afferent responses to rotation [80], as well as periodic fluctuations in background discharge of irregular afferent fibres [81]. This irregular pattern consists of both fast and slow response components and has also been reported in monkeys [5, 18], and cats [6]. Leijon and Magnusson (2014) suggest that the distinctive discharge pattern may modulate the sensitivity of irregular afferents in response to the fast and slow components of cupula activation during abrupt accelerations. Despite this, the purpose of this afferent modulation remains unclear. An attractive early hypothesis was that vestibular efferent activity modulates afferent discharge during volitional head movements—although Cullen and Minor (2002) found no difference in vestibular efferent modulation of vestibular afferent discharge between passive and active head movements in alert macaques. While from this observation it seems unlikely that vestibular efference simply differentiates between active and passive movements, it is reasonable to suggest that based on the diversity of inputs to the EVN [70], and the heterogeneity of synaptic profiles shown here, the EVN is suited to the modulation of peripheral sensitivity in other context-dependent ways. For example, during states of arousal or predation [39, 82], or under conditions that stimulate aspects of autonomic nervous system response, including cardiovascular regulation [83] and stress [84].

## Conclusions

Effective interaction with our environment is dependent on our ability to maintain equilibrium. Previous work describing the central control of the peripheral vestibular organs have primarily focused on end organs and afferent fibre discharge, while little attention has been afforded to the intrinsic and/or synaptic properties of the efferent neurons that modulate them. The present study is amongst the first systematic investigations of the intrinsic and synaptic influences on efferent vestibular nucleus neurons in any species. In contrast to neighbouring vestibular neurons, EVN neurons display a distinctive homogeneous discharge pattern and receive heterogeneous synaptic inputs that make it possible that the functional role of these neurons may be to signal changes in stimuli context; i.e. modulation of the sensitivity of vestibular periphery in a state-dependent manner.
